# Understanding the Role of Oxidative Stress, Neuroinflammation and Abnormal Myelination in Excessive Aggression Associated with Depression: Recent Input from Mechanistic Studies

**DOI:** 10.3390/ijms24020915

**Published:** 2023-01-04

**Authors:** Anna Gorlova, Evgeniy Svirin, Dmitrii Pavlov, Raymond Cespuglio, Andrey Proshin, Careen A. Schroeter, Klaus-Peter Lesch, Tatyana Strekalova

**Affiliations:** 1 Laboratory of Psychiatric Neurobiology, Institute of Molecular Medicine and Department of Normal Physiology, Sechenov First Moscow State Medical University, 119991 Moscow, Russia; 2 Laboratory of Cognitive Dysfunctions, Institute of General Pathology and Pathophysiology, Russian Academy of Medical Sciences, 125315 Moscow, Russia; 3 Neuroplast BV, 6222 NK Maastricht, The Netherlands; 4 Hotchkiss Brain Institute, Alberta Children’s Hospital Research Institute, University of Calgary, Calgary, AB T2N 4N1, Canada; 5 Centre de Recherche en Neurosciences de Lyon (CRNL), 69500 Bron, France; 6 P.K. Anokhin Research Institute of Normal Physiology, 125315 Moscow, Russia; 7 Preventive and Environmental Medicine, Kastanienhof Clinic, 50858 Köln-Junkersdorf, Germany; 8 Department of Psychiatry and Neuropsychology, School for Mental Health and Neuroscience (MHeNs), Maastricht University, 6229 ER Maastricht, The Netherlands; 9 Division of Molecular Psychiatry, Center of Mental Health, University Hospital Würzburg, 97080 Würzburg, Germany

**Keywords:** major depressive disorder (MDD), aggression, neuroinflammation, oxidative stress, insulin receptor, myelination

## Abstract

Aggression and deficient cognitive control problems are widespread in psychiatric disorders, including major depressive disorder (MDD). These abnormalities are known to contribute significantly to the accompanying functional impairment and the global burden of disease. Progress in the development of targeted treatments of excessive aggression and accompanying symptoms has been limited, and there exists a major unmet need to develop more efficacious treatments for depressed patients. Due to the complex nature and the clinical heterogeneity of MDD and the lack of precise knowledge regarding its pathophysiology, effective management is challenging. Nonetheless, the aetiology and pathophysiology of MDD has been the subject of extensive research and there is a vast body of the latest literature that points to new mechanisms for this disorder. Here, we overview the key mechanisms, which include neuroinflammation, oxidative stress, insulin receptor signalling and abnormal myelination. We discuss the hypotheses that have been proposed to unify these processes, as many of these pathways are integrated for the neurobiology of MDD. We also describe the current translational approaches in modelling depression, including the recent advances in stress models of MDD, and emerging novel therapies, including novel approaches to management of excessive aggression, such as anti-diabetic drugs, antioxidant treatment and herbal compositions.

## 1. Major Depressive Disorder and Excessive Aggression

Major depressive disorder (MDD) is one of the most widespread and debilitating mental disorders, but its molecular aetiology remains poorly understood. Currently, the diagnosis of MDD, according to the *Diagnostic and Statistical Manual of Mental Disorders* (5th edition), is determined by two or more weeks of depressed mood and/or loss of interest and pleasure (anhedonia), along with such symptoms as changes in sleep, weight and various accompanying emotional abnormalities, including anxiety, agitation, psychomotor inhibition and aggression [1]. Over the last decade, a significant increase of almost 20% has been reported in the incidence of MDD [2], with depressive disorders now considered as severe and disabling diseases that affect more than 300 million people worldwide, along with a proportional increase in patients prone to aggression and violence [3,4]. Aside from mental health issues, depression is known to be a serious life quality aggravation factor in patients, diminishing not only emotional state but also somatic health [5].

The modern term “depression” is associated with a prevalent feeling of sadness together with the inability to experience pleasure and deficits in daily functioning [1]. Depression covers a wide range of psychopathological manifestations of mood disorders that vary in typological structure, severity and duration. Our main focus in this review is agitated depressive disorder, which is characterized by an inner psychotic agitation coupled with deficient impulse control and aggressive behaviour. Generally, when aggression in adults is not a response to a clear threat, it is considered a sign of mental disorder [6]. The comorbidity between aggression and MDD, among other mental illnesses where psychiatric disorders may be associated with violence, has been demonstrated in numerous studies [4,7,8,9,10]. Anxiety- or aggression-driven depression has even been proposed as a subtype of MDD, in which aggression dysregulation is not only a symptom but also a pacemaker of disorder progression [11].

Importantly, while a century ago MDD was typically characterized by psychomotor inhibition and retardation, in the last two decades approximately one-third of depressed patients demonstrated excessive aggression and anger attacks [12], with almost 40% of patients now registered [4]. As such, the proportion of depressed patients with the agitated form of the disease and symptoms of aggression and violence is rapidly increasing. Meanwhile, the generally accepted therapy for MDD is not fully oriented to meet the modern clinical features of this disease and the frequent incidence of symptoms of aggression and agitation does require new therapies that go beyond standard antidepressant treatment. As treatment with antidepressants is often indicated, ~50% of patients do not achieve remission with first-line treatment [13]. Moreover, commonly used antidepressants were shown to exacerbate symptoms of aggression and suicidality, which is considered to be a form of self-aggression that is particularly frequent in adolescent patients with depression [14,15,16]. This indicates the need for the development of more effective and safe treatments based on an in-depth understanding of MDD’s pathophysiology when accompanied by agitation and aggression.

Extensive studies at different molecular levels point to a high complexity of numerous interrelated pathways as the underpinnings of MDD and its highly heterogeneous symptomatology, including agitated depression with manifestations of excessive aggression [17,18]. Major systems under consideration include monoamines, the hormonal axis of stress response, neurotrophins, excitatory and inhibitory neurotransmission, mitochondrial dysfunction, epigenetics, inflammation, the opioid system, myelination and the gut–brain axis, among others [17,19]. Currently, a vast body of the latest literature points to new mechanisms for MDD and its associated symptoms, such as impulsivity and aggression. Here we overview these key mechanisms, which include neuroinflammation, oxidative stress, insulin receptor (IR) signalling and abnormal myelination, and discuss the hypotheses that integrate these processes as the neurobiological basis of MDD. We also discuss management of excessive aggression using novel emerging therapies such as antioxidant and herbal composition treatments and anti-diabetic drugs.

## 2. Animal Models of Excessive Aggression

The use of animal models is the key to investigating the mechanistic aspects of MDD. Maladaptive aggressive behaviour, manifesting as aggressive behaviour that exceeds species-typical levels or patterns, is defined in rodents by a short latency to initiate the attack, long duration of and high intensity of attacks potentially leading to an injury, lack of species-normative behavioural structure and insensitivity to behavioural signals of submission [20]. Various genetic and environmental factors, such as stress and social threat, can be used to induce experimental aggression in the available animal models (see Table 1).

The role of genetic factors in excessive aggression can be investigated with the use of transgenic animal models. Reduced scores of aggression have been found in male knockout mice lacking the long form of the dopamine D2 receptor [21,22], the α-isoform of the oestrogen receptor [23,24], the arginine vasopressin V1b receptor [25] or dopamine β-hydroxylase [22,26]. Elevated aggression was shown in mice lacking tryptophan hydroxylase 2 (Tph2) [27,28], serotonin (5-HT) 1B receptor [29], dopamine transporter (DAT) [30], nitric oxide synthase (NOS) [31] and monoamine oxidase A (MAOA) [32].

One common approach for excessive aggression is the selective breeding of mouse and rat strains for high aggression scores. A commonly used model is the mouse line bred for short attack latency (SAL); such mice display elevated aggression correlating with low brain 5-HT levels and reduced reuptake transporter activity [33,34]. Excessive and abnormal forms of aggression are also demonstrated in Turku Aggressive mice [35] and North Carolina 900 and North Carolina 100 mice [36]. Selectively bred Wistar rats with low anxiety-like behaviour (LAB), initially used as controls for rats with high anxiety-like behaviour (HAB), also display high and abnormal forms of aggression [37,38]. The prairie vole (*Microtus ochrogaster*) has been proposed as an animal model for investigating the neurobiology of escalated aggression and violence, since ethological mating of these mice is accompanied by aggressive behaviour directed toward both male and female conspecifics [39].

Stress, a well-known factor for MDD, may elicit aggression and accompanying behavioural abnormalities in rodents. Various stressful conditions are generally used to provoke elevation in aggressive behaviour in conventional mouse lines [40]. One of the most-used manipulations to provoke excessive aggressive behaviour in male mice is social isolation [41]. Socially isolated mice can demonstrate aggressive behaviour in the resident–intruder test [42,43], which was found to be accompanied by alterations in the function of the hypothalamic-pituitary-adrenal (HPA) axis, suggesting the stressful nature of isolation in some mouse strains [44]. Maternal separation stress, a commonly accepted risk factor for MDD, was shown to have both a short- and a long-term effect on aggression. In rats, maternal separation during the first two weeks of life significantly increased intermale aggression at 14–16 weeks of age and lowered maternal aggression [45]. Interestingly, in mice, this experimental procedure of maternal separation stress was shown to reveal gender differences in intermale and maternal aggression; maternally separated females tended to be more aggressive towards male intruders than control females, whereas in males, maternal separation decreased intermale aggression [46]. Post-weaning social isolation or subjugation also caused elevated intermale aggression in mice and rats, as well as in hamsters and guinea pigs [47].

**Table 1:** Animal models of aggression associated with depressive syndrome. This summary provides a neurobiological classification of animal models of aggression associated with depressive syndrome starting with an impact of a single gene out to varieties of environmental factors. Core characteristics highlight the important role of monoamines, pro-inflammatory shifts and oxidative stress in the development of a valid animal model that recapitulates behavioural phenotypes of increased aggressiveness associated with pro-depressant changes. Abbreviations are: BDNF—brain-derived neurotrophic factor; GSK3-β—glycogen synthase kinase-3 beta; GABA—gamma-aminobutyric acid; IRS-1—insulin receptor substrate-1; IRS-2—insulin receptor substrate-2; 5-HT—5-hydroxytryptamine (serotonin); ERα—α-isoform of the oestrogen receptor; D2Rß dopamine D2 receptor; V1bR—vasopressin 1b receptor.

Animal Model	Strains	Core Characteristics	References
**Genetic Models**			
Knock-out dopamine D2 receptor	D2R^−/−^ mice	Elevated aggression in males, reduced hypothalamic orexin precursor expression, increased serum prolactin levels	[21,48]
Knock-out α-isoform of the oestrogen receptor	ERα^−/−^ mice	Elevated aggression in males, compromised neuroplasticity	[23,24]
Knock-out arginine vasopressin V1b receptor	*V1bR*^−/−^ mice	Elevated aggression in males, compromised neuroplasticity, decreased neurogenesis	[49]
Knock-out dopamine β-hydroxylase	DBH^−/−^ mice	Elevated aggression in males, compromised neuroplasticity, decreased insulin receptor substrate-1 (IRS-1) and insulin receptor substrate-2 (IRS-2) signalling	[26,48]
Knock-out tryptophan hydroxylase 2	Tph2^−/−^ male mice	Elevated aggression in males, decreased 5-HT level, compromised neuroplasticity	[27,28]
Knock-out 5-HT1B receptor	5-HT1B^−/−^ mice	Elevated aggression in males, deficient neuroplasticity	[29]
Knock-out dopamine transporter	DAT^−/−^ mice	Elevated aggression in males, deficient synaptic plasticity	[30]
Knock-out nitric oxide synthase	NOS^−/−^ mice	Elevated aggression in males, compromised neuroplasticity, antioxidant system disbalance	[31]
Knock-out MAOA	MAOA^−/−^ mice	Elevated aggression in males, deficient synaptic plasticity and pruning, disbalance of brain monoamine levels	[32]
Bred for short attack latency (SAL)	SAL mice	Elevated aggression, low brain 5-HT level, reduced 5-HT reuptake transporter activity	[33,34]
Turku Aggressive mice	Turku Aggressive mice	Elevated aggression in males	[35]
North Carolina 900 mice	NC900 mice	Elevated aggression in males, reduced GABA-ergic neurotransmission	[36]
North Carolina 100 mice	NC100 mice	Elevated aggression in males, lower dopamine concentrations	[36]
Wistar rats with low anxiety-like behaviour (LAB)	LAB rats	Elevated aggression in males, compromised neuroplasticity	[37,38]
**Environment Stress Models**			
Social isolation	CD1, C57BL/6J mice	Excessive aggressive behaviour in males, alterations in the function of the HPA axis	[41,42,43,44]
Maternal separation	C57BL/6J mice	Rats: increased intermale aggression at 14–16 weeks of age, lowered maternal aggression. Mice: females are more aggressive towards male intruders; males are less aggressive towards male intruders	[45,46]
Chronic mild stress	BALB/C, CD1, C57BL/6J mice	Increased offensive and aggressive behaviours in males; GSK3-β overexpression; microglial activation, reduced neuroplasticity	[18,50,51,52,53,54,55]
Rat exposure	C57BL/6 mice	Increased aggressive behaviour in males, aberrant neurogenesis, reduced neuroplasticity; oxidative stress	[52,56]
Social defeat	C57BL/6 mice	Excessive aggression in dominant males, microglial activation, reduced neuroplasticity and synaptic pruning, deficient neurogenesis, GSK3-β overexpression, oxidative stress	[57,58,59,60,61]
Ultrasound stress	BALB/C, CD1, C57BL/6J mice; Wistar, Sprague-Dawley rats	Increased aggressive behaviours in males; microglial activation, reduced neuroplasticity, GSK3-β overexpression, oxidative stress	[62,63,64,65,66,67]
**Maternal Models**			
Stimuli from pups	BALB/C, CD1, C57BL/6J mice; Wistar, Sprague-Dawley rats; Syrian hamsters;	Increased aggressive behaviours in females, deficient neuroplasticity and reduced neurogenesis	[68,69,70,71]
**Gene × Environment Interaction Models**			
Deficiency of tryptophane hydroxylase and 5-day predation stress	Tph2^+/−^ male mice	Increased aggressive behaviours in males; reduced brain serotonin content, reduced expression of 5-HT6 receptor, GSK3-β overexpression	[72]
	Tph2^+/−^ female mice	Increased aggressive behaviours in females; reduced brain serotonin content, GSK3-β and myelin basic protein overexpression; deficient neuroplasticity, downregulation of synaptophysin	[73]

Another stress paradigm that is used to elicit aggression in experimental rodents is chronic mild stress [18,74]. Chronic unpredictable stress was found to provoke increased aggression in male BALB/C, CD1 and C57BL/6J mice, as shown in the resident-intruder test [51,52,53,54,55]. C57BL/6J mice exposed to a chronic mild stress paradigm showed increased offensive and aggressive behaviours in the resident-intruder test and the social dominance tube test [75]. Rat exposure, which is an ethologically valid stressor because rats are natural predators of mice, caused an elevation of aggressive behaviour in male C57BL/6 mice [52,56]. The social defeat paradigm has also been shown to induce excessive aggression in dominant males 2 [61]. Among the chronic stress paradigms, the ultrasound stress procedure has attracted growing attention from researchers as the model of “emotional” stress in rodents [65,66,67]. In humans, this corresponds to emotional neglect, loss of a parent or child abuse, and may contribute to various psychiatric disorders, including the development of MDD, violence and abnormal aggression [76,77]. “Emotional stress” is generally seen as a form of stress evoked by processing a negative mental experience rather than an organic or physical disturbance [78] and is commonly regarded as a human-specific trait that is challenging to model in other species. However, recent studies demonstrated that a 3-week-long exposure to ultrasound of unpredictable alternating frequencies within the ranges of 20–25 and 25–45 kHz can induce depression-like characteristics in laboratory mice and rats, which are accompanied by increased aggressive behaviour sensitive to pharmacotherapies [64,65,66].

Indeed, “emotional stress”, which is referred to as a state that is primarily triggered by the perception and cognitive evaluation of adverse events rather than disturbance of a physical nature, appears to be the type of stress that frequently results in overt aggressiveness in many studies [79,80]. Specifically, a recently established model of emotional stress with “emotionally negative” and “neutral” randomly alternating frequencies of ultrasound in the range 20–45 kHz [64,81] for three weeks resulted in increased aggressive, depressive-like and anxiety-like behaviours in stressed mice, accompanied by elevated oxidative stress, neuroinflammation and disrupted neuroplasticity [62,63,66,67]. The comorbid nature of depressive-like changes and increased aggressive behaviour in the ultrasound stress model has been proven using several mouse and rat strains [62,64,66,67,82].

Excessive aggression can also be modelled in rats by the administration of glucocorticoids, which mimics the hormonal component of stress exposure [83,84]. Apart from stress models, escalated aggression can be induced in animal models of experimental alcohol addiction during withdrawal from prolonged exposure to repeated high alcohol doses, which is modelled in mice, rats and monkeys, or by acute alcohol exposure [85]. This model is of particular relevance, as in humans, aggression is heavily associated with use of alcohol [86] which also can further aggravate depression [87,88]. According to the World Health Organization, alcohol consumption is more strongly associated with aggressive behaviour than the use of any other psychotropic substance [89], with alcoholics being 2–3 times more likely to experience depressive symptoms than the average population [88]. Absence of an expected dietary reward can also induce a state of hyper-aggression [90].

There is a significant body of evidence indicating a combined contribution of genetic background and aversive life experiences during childhood, adolescence and adulthood to the development of MDD associated with elevated aggression and antisocial behaviour [91]. Family studies of aggressive behaviour suggest that, in both males and females, 50% of the variance in aggressive behaviour parameters can be explained by environmental factors 1 [92]. Such aversive experiences include emotional stress, evoked by processing a negative mental experience, which is a state primarily triggered by the perception and cognitive evaluation of adverse events [78]. This clinical situation requires the use of appropriate animal models mimicking gene × environment (G × E) interactions in rodents. As such, the use of animal models of “emotional stress” can be of particular interest in the context of modelling such conditions. Recently, increased aggressiveness was reported in mice with a partial deficiency of the gene encoding tryptophane hydroxylase 2, a key enzyme for 5-HT synthesis (Tph2^+/−^ mice), after their exposure to a 5-day predation [72,93]. Predation stress used in such mutants that do not display any behavioural alterations under normal conditions was used as an analogue of “emotional” stress in mice because it did not imply any physical challenges to an animal except the visual and olfactory cues of a rat. Brain tissue concentrations of serotonin, its precursor 5-HT and its metabolite 5-hydroxyindoleacetic acid were significantly altered for all groups in the prefrontal cortex (PFC), striatum, amygdala, hippocampus and dorsal raphe after stress of male mutants [72]. Compared to non-stressed animals, the concentration of 5-HT was elevated in the amygdala but decreased in the other brain structures. Overexpression of the AMPA receptor subunit, GluA2, and downregulation of the 5-HT6 receptor, as well as overexpression of c-Fos and glycogen-synthase-kinase-3β (GSK3-β), were found in most structures of the stressed Tph2^+/−^ mice [72].

Thus, models utilizing emotional stress and gene × environment interaction might provide high aetiological validity for modelling aggression associated with stress, an etiological factor of MDD, and neuropsychiatric pathologies in general. This is due to the fact that emotional or psychological stress is the most frequent form of stress in humans; therefore, these models may be a promising way to investigate the neurobiology of excessive aggression associated with a depressive-like state and possible further treatments.

Finally, several types of animal models of female aggression, which is a special domain in the field of neuropsychiatric translational research, were also proposed. Historically, rodent models of maternal aggression (i.e., defensive behaviour against a potentially dangerous intruder that is intended to protect the offspring) were proposed first. In mice and rats, such aggression is triggered by stimuli from the pups (i.e., suckling stimulus) and the presence of an intruder [68,69]. However, rodent maternal aggression is less applicable for modelling human psychiatric pathology. Furthermore, social aggression, such as intermale territorial aggression, is much less common in female mice [94]. Recently, the prairie vole (*Microtus ochrogaster*) has emerged as a new animal model for investigating the neurobiology of escalated aggression and violence because, ethologically, their mating is accompanied by aggressive behaviour directed toward both male and female conspecifics [39]. Another highly recognized model of female territorial aggression is the Syrian hamster (*Mesocricetus auratus*), because in this species both males and females are highly territorial and females tend to be aggressive and dominant over male intruders [70,71]. However, although there are rodent models of female aggression that mimic ethologically relevant behaviours, little research is directed towards modelling female aggression in pathology, including MDD.

Recently, Tph2^+/−^ mice exposed to predator stress were shown to display excessive aggression, increased anxiety-like behaviours, and altered sociability and compromised brain metabolism of dopamine and noradrenaline [95,96]. The predation stress procedure elicited behavioural and molecular changes in Tph2^+/−^ mice that were the opposite of those observed in control mice [73,95]. As such, environmentally challenged Tph2^+/−^ mice may represent a valid model of aggression that recapitulates the role G × E interaction in the mechanisms of stress-related aggression.

Thus, while a large variety of animal models of aggression are currently available, it can be suggested that those utilizing the “emotional stress” paradigm and modelling G × E interaction are likely to be the most promising experimental approaches to modelling excessive aggression associated with depressive symptoms.

## 3. Neuroanatomical Basis of Aggression in Humans and Rodents in the Context of Depressive Disorder

Despite the fact that there is still no clear understanding of specific neuroanatomical connections that underlie both depression and excessive aggression, many brain regions were shown to be implicated in these abnormalities [97,98]. Aggression comprises a suite of agonistic behavioural interactions; thus, various species-specific relevant signals, including danger and emotional stressors, are transduced by sensory afferents to the central nervous system (CNS) where this information is processed by the limbic neurocircuitry [99,100]. Data from animal and human studies suggested several key brain regions, primarily in the limbic system, associated with aggression and depressive syndromes [101].

The amygdala is generally regarded as a key brain structure regulating aggressive behaviour [101,102,103]. It mediates fear and defensive responses [104] and is important in the processing of emotionally adverse events [105]. In early studies, patients with damage to the amygdala demonstrated impairment in the recognition of fearful facial expressions [106]. Children with conduct disorders and prominent aggressive behaviour generally have smaller prefrontal cortex, amygdala and hippocampus volume [107]. In mice, inter-male aggression-related behaviours were inhibited following medial amygdala lesioning [108]. Neurons of medial amygdala are active during social behaviours such as fighting and mating [109].

The dorsolateral prefrontal cortex and orbitofrontal cortex receive inputs from the amygdala and other medial temporal areas that may integrate sensory information with affective signals [110]. The available literature proposes a pivotal role for the amygdala in the network between these structures that underlies the processing of emotional and goal directed behaviour, and the dysfunction of any of these structures results in problems with the regulation of emotion. This can manifest as difficulties with the inhibition of aggressive behaviour [111,112], with violent patients often having reduced prefrontal–amygdala and prefrontal–striatal connectivity [113]. Thus, an imbalance between the regulatory influence of the prefrontal cortex and the responsivity of the amygdala is chiefly implicated in excessive aggression [114].

Studies with early gene c-Fos in animal models of environmental stress and fMRI in humans confirmed important roles for the prefrontal cortex and hippocampus, the medial preoptic area, anterior, lateral and ventromedial hypothalamus, medial and central amygdala, the locus coeruleus, bed nucleus of the stria terminalis, dorsal raphe and the periaqueductal grey matter [99]. There is a large overlap among the brain areas involved in different types of aggression, but some peculiar differences also exist, such as maternal aggressive behaviour and male escalated aggressive behaviour [111]. In particular, variable levels of escalated aggression were shown to be associated with the changes in the activity in the brain structures, which may be either elevated or decreased. Specifically, the periaqueductal grey matter in the midbrain was found to be hyperactivated because this region is normally activated during inter-conspecific aggression. However, the degree of its activation decreases in animals genetically selected to display higher aggressive behaviour at the baseline levels [99]. By using c-Fos staining as a marker of neuronal activation, it was shown that agonistic encounters result in different activation patterns in LAL and SAL mice [99]

Studies with lesions or optogenetic manipulations have confirmed the involvement in aggressive behaviour of the aforementioned brain regions, together with other brain structures, such as the cerebellum [22,115]. Optogenetic bidirectional manipulation with neuronal activity has helped to elucidate the role of increased glutamate signalling and decreased GABA neurotransmission in the midbrain and cortical structures, along with the role of increased activity of vermis Purkinje cells in the cerebellum, in the mechanisms of escalated aggressive behaviour [115,116]. In addition, cortical or injuries lesions of these CNS structures were shown to result in disinhibited aggressive behaviour [117]. Prefrontal grey matter is suggested to be implicated in the mechanisms of impulse control and behaviour, as individuals with antisocial personality disorder display volume reductions in this area of the brain [118,119]. Furthermore, clinical studies have demonstrated that patients with impulsive aggression were shown to have lower metabolic activity in the prefrontal cortex [120].

As mentioned above, neural circuits that modulate aggressive behaviour also include the hippocampus and hypothalamus [121,122,123,124,125]. Specifically, atypical hippocampal anatomical asymmetries that disrupt prefrontal–hippocampal circuitry may result in emotion dysregulation with increased aggressiveness because hippocampal neurons have robust projections that originate from hippocampal CA1 and terminate in the orbital and medial frontal cortices [126]. In mouse studies, stress-induced attacking behaviour was shown to be associated with neuronal activation of the ventral hippocampus [126]. Stimulation of pyramidal neurons in the CA2 region of the hippocampus, which are important for social memory, promotes social aggression in mice [123].

Electrical stimulation of the ventromedial nucleus and lateral hypothalamus can elicit aggressive behaviour in animals [127,128]. In these studies, electrical stimulation induces an extreme aggression that can be directed against both genders and even dead animals, highlighting the pathological nature of a hypothalamus-mediated aggressive behaviour [129,130]. The ventromedial nucleus receives inputs from the lateral hypothalamus as well as the cortical and basolateral amygdala, which modulate the expression and duration of aggressive behaviours [131]. The central, lateral and basal nuclei of the amygdala facilitate aggressive attacks [132,133] and their effects are associated with the overexpression of glutamatergic GluR1 receptors, which is the opposite for the prefrontal cortex [22]. Human studies also suggest that the hypothalamus is related to the control of aggressive behaviour [134,135]. Neuroimaging fMRI studies showed the hyperactivity of the hypothalamus in aggressive individuals and in domestic violence offenders [136].

Thus, the neuroanatomical substrate of aggression associated with depressive syndrome itself represents a complex network that can vary greatly, depending on the type of aggressive behaviour, features of depression in an individual patient, age and gender.

## 4. Neuroinflammatory Mechanisms of Depression and Excessive Aggression

Traditionally, the monoamine hypothesis is implicated in the vast majority of behavioural abnormalities associated with pro-depressant behavioural changes [137], and early models of aggression associated with depressive syndrome are largely focused on the role of monoamine neurochemical deficits [138]. In support of this hypothesis, it has to be stated that monoamine inhibitors (MAOI) are effective as antidepressants that also lower the aggressiveness level by increasing serotonergic and noradrenergic signalling [138]. Later, the monoamine hypothesis has emphasized the role of deficits in dopaminergic signalling for triggering both anhedonia and impulsive aggression [139]. However, important limitations of the monoamine hypothesis and other neurochemical deficit models were also apparent: not all drugs that modulate monoaminergic signalling are effective modulators of aggression or antidepressants [137]. Furthermore, some selective 5-HT reuptake inhibitors (SSRIs) were reported to induce aggression in depressed patients [14]. Generally, aggression and depression are widely understood to be heterogeneous phenomena with a weak correspondence to any single biological substrate [140].

Nowadays, the implication of the immune system in the pathophysiology of aggression associated with depressive syndrome is being recognized [102,141,142]. The diverse collection of immune cells and non-cellular factors profoundly influences most aspects of the stress response and the pathophysiology of depressive syndromes and their comorbidities, including agitation and aggressiveness. Patients experiencing emotional stress display long-term changes of brain glial cells: activated microglial cells with less ramified and shortened processes [143,144]. These changes are hallmarks of both female and male pathophysiology of MDD and have been replicated in animal models of depression [145]. Activated microglia and reactive astrocytes have been shown to produce pro-inflammatory cytokines and to stimulate other immune cells to produce cytokines and inflammasomes as their response to neuronal activation triggered by emotional stress [145]. Notably, both an aggressive experience and the expectation of an aggressive event are associated with increases in inflammatory cytokines, which can be the result of sympathetic activation and HPA axis activation [146,147]. Aside from stress which contributes to glial activation, neuroinflammation may be caused by such factors as viral or bacterial diseases of the CNS [148,149,150], as well as systemic inflammation caused both by infection [151] or aseptic condition, as in case of type 1 and 2 diabetes [152]. Alimentary factors, e.g., “Western diet” [153,154], as well as environmental toxicity, e.g., metal toxicity, may also contribute to neuroinflammation [155].

Human studies show that individuals with excessive aggression display elevated inflammatory cytokine levels and dysregulated immune responses in the CNS and in blood, and comorbidity of depression and aggression is correlated with stronger immune dysregulation [141]. Elevated aggressive traits were associated with increased serum tumour necrosis factor (TNF) [156] and the inflammatory marker C-reactive protein (CRP) [157]. CRP has been suggested as a predictor of the risk of aggressive behaviour among psychiatric inpatients [158]. It has been reported that immunotherapy to treat patients with hepatitis C by chronic administration of interferon alpha (IFN-α) increases irritability and anger/hostility in some patients [159]. Furthermore, pro-inflammatory cytokines IL-2 and TNF, along with anti-inflammatory factors IL-4, IL-5 and IL-10, were significantly elevated in patients with excessive aggression and post-traumatic stress disorder who underwent early life trauma [160]. Married couples show increases in plasma IL-6 and TNF after conflict interactions, and these increases in cytokines were larger in couples who showed more hostile behaviour during their conflict interactions [161]. IL-6 levels were also higher in subjects with intermittent explosive disorder [162].

Pre-clinical translational studies are keeping up with these clinical observations. Wild type C57BL/6 mice bred for high aggression had increased cytokine levels, with knockout of both TNF receptors R1 and R2 resulting in the absence of aggressive behaviour [163]. Deletion of TNF receptors R1 and R2 reduced the duration of aggressive behaviours in the resident–intruder test in male mice, which is in line with findings from human studies in which serum TNF is increased in highly aggressive individuals [163]. Glycogen synthase kinase-3 (GSK-3), which is closely related to pro-inflammatory cytokine regulation, has been demonstrated to promote inflammation, as well as aggressive and depression-like behaviours in rodents, whereas reduced expression of either GSK-3 isoform results in decreased aggressive behaviours in mice [66,67,164].

Thus, both clinical and animal studies cumulatively suggest the prominent role of inflammation and immune dysregulation in the pathophysiology of aggression in depressed patients.

## 5. Oxidative Stress Markers, Insulin Receptor Signalling and Excessive Aggression

Inflammation is closely related to the increased excessive formation of free radicals in mitochondria, known as oxidative stress [165,166], which can play a key role in the pathophysiology of emotional stress, depression and accompanying behavioural abnormalities, including excessive aggression [167,168]. Oxidative stress markers have been found to be elevated in alcohol-induced aggressive, impulsive and suicidal behaviour [169]. More specifically, human studies suggest a positive relationship between plasma markers of oxidative stress–8-hydroxy-2’-deoxyguanosine (8-OHdG) and 8-isoprostane–and aggression in human subjects [170]. These markers were elevated in adult subjects with personality disorder who displayed aggressive traits [171]. At the same time, the meta-analysis revealed that 8-OHdG and F2-isoprostanes, mirroring oxidative DNA and lipid damage, respectively, were increased in depressed patients [172]. A significantly elevated level of serum malondialdehyde was found in those patients with major depression displaying aggression compared to healthy controls [173].

In animal studies, measures of oxygen radicals in Long-Evans rats were shown to correlate with their aggression scores [174], and the intracellular redox status of peripheral blood granulocytes correlated significantly with the aggressive behaviour levels of adult male mice [175]. Impaired antioxidant defence can also have a direct effect on aggressive behaviour. Mice deficient in copper–zinc superoxide dismutase (SOD1) that express 50% of this antioxidant enzyme are more aggressive than wild-type males [176]. A depressive-like state in mice induced by repeated restraint stress was associated with upregulation of NADPH oxidase and the resulting metabolic oxidative stress, whereas inhibition of NADPH oxidase provides beneficial antidepression effects [177]. Acute restraint stress is also known to induce depressive-like and aggressive behaviour in rodents and is reported to cause neuronal oxidative damage in mice, reducing catalase and superoxide dismutase activity in the brain. Depressive-like behaviour in mice caused by repeated glucocorticoid administration caused a decline in the antioxidant defence system, as shown by the reduced glutathione levels [178]. In the ultrasound model of “emotional stress”, BALB/c mice demonstrated aggressive behaviour accompanied by increased concentrations of protein carbonyl and total glutathione [66], as well as of malondialdehyde [62]—markers of oxidative stress—in the prefrontal cortex and hippocampus.

In summary, oxidative stress appears to be an important pathophysiological mechanism underlying both the depressive-like state and excessive aggression. It is believed that mitochondrial dysregulation and microglia activation associated with oxidative stress can lead to neuronal dysfunction, compromised brain plasticity [179,180] and also demyelination [181], which underlies deficits in brain connectivity and impulse control. Consequently, this deficient impulse control can lead to increased aggressiveness. Indeed, increased concentrations of oxidative stress markers are suggested to result in damage to the periventricular white matter, with a paucity of mature oligodendrocytes and hypomyelination [182]. Increased oxygen species may impair oligodendrocyte precursor cell proliferation and differentiation, resulting in disrupted myelination [183]. Increased activation of oxidative stress pathways was found to slow down endogenous white matter repair by disrupting the renewal processes [184].

Furthermore, elevated oxidative stress was shown to interfere with IR functioning, the signalling of which is a well-established mechanism implicated in the pathophysiology of depression, and can regulate inflammation and myelination [185,186]. For example, reactive oxygen species such as H_2_O_2_ have been established as a triggering events of IR-mediated signalling associated with development, neuroprotection, metabolism and plasticity in the brain and the resulting behavioural changes, including depressive disorder [187].

Compounds stimulating IR functions were shown to decrease the manifestations of stress-induced depressive and aggressive behaviours [188,189]. A new class of compounds called “insulin receptor sensitizers” were investigated for their preclinical and clinical efficacy in depressed patients. For example, recent clinical and translational studies have revealed antidepressant-like effects, increased mitochondrial biogenesis in neurons and decreased neuronal damage and anti-inflammatory effects for the thiazolidinediones rosiglitazone and pioglitazone, which can potentiate the binding of insulin to the IR [182]. Thus, further clinical studies of IR sensitizers can be promising in normalizing IR functions and associated behavioural changes, including excessive aggression.

## 6. Role of Disrupted Myelination and Connectivity in Excessive Aggression and Impulsivity Associated with Depressive Syndrome

The recent literature suggests that neuroinflammation and oxidative stress may underlie many pathological processes in the nervous system that can be partially mediated via the affected CNS myelination [190,191,192]. Myelination is one of the major postnatal CNS developmental milestones that ensures efficient neuronal circuit connectivity [191]. Myelin sheaths are electrically insulating structures consisting of a lipid-rich substance wrapped around axons in both the CNS and the peripheral nervous system [193]. For a long time, it was thought that the main functions of myelination are to increase maximum conduction velocity and decrease axonal energy consumption.

However, a growing body of evidence suggests that myelinating oligodendrocytes are involved in other processes, such as supporting axonal energy metabolism via myelin sheaths [192,194,195]. In contrast to what was thought earlier, it is now established that myelination is not a single developmental event but rather a constant process of de novo formation of myelin in the nervous system [196]. Remodelling of the myelin sheaths, which was shown to be dependent on neural activity, is now thought to be involved in learning and long-term neuroplasticity [197] and the stress response [93,96]. For example, it was shown that in mice, fear learning induces oligodendrocyte precursor cell (OPC) proliferation and differentiation into myelinating oligodendrocytes in the medial PFC, whereas in transgenic mice with conditional deletion of Myrf transcription factor in OPCs, which prevents OPC maturation and expression of myelin structural genes while preserving existing myelin sheaths, long-term fear memory retrieval is impaired [198].

Deficits in myelination are observed in neurodevelopmental conditions such as autism spectrum and attentiondeficit/hyperactivity disorders [199,200,201] and schizophrenia [202]. In addition, in clinical studies, prenatal SSRI exposure or social isolation, which are detrimental for CNS development and function, were shown to be associated with abnormalities in myelination [203] Post-mortem pathological assessments of patients with depression have revealed the reduction in myelination in regions of the limbic system of the brain, such as the prefrontal cortex [204], hippocampus [205] and striatum [206].

Social isolation in juvenile mice, which is known to cause depressive-like and pro-aggressive behavioural changes, led to hypomyelination in the medial prefrontal cortex and lowered activation of this brain structure during exposure to a stranger counter-partner mouse, while re-socialization reverted the hypomyelination [207]. Psychological stress (e.g., chronic social defeat stress) was also shown to affect myelination in a strain-specific and region-specific manner. Stress-susceptible B6 mice showed thinner myelin sheaths in the ventral prefrontal cortex and downregulation of myelination genes in the medial PFC, whereas stress-resilient mice had thicker myelination in the medial prefrontal cortex [208].

Studies with other rodent models, such as chronic social defeat stress [209] and unpredictable chronic mild stress [210,211], showed impairment of oligodendrocyte differentiation and also myelination in the brain. With myelination being crucial for brain connectivity and signal propagation, dysmyelination may cause impairment of connections between structures involved in the regulation of aggression. For example, such changes can contribute to aggressive and impulsive behaviours via disruption of cortical–subcortical connectivity—so-called “top-down” behavioural control. Increased aggression accompanies impairments in medial prefrontal cortex, such as amygdalar dysconnectivity, which can be hypothesized to arise from insufficient estimation of the possible consequences of engagement in impulsive aggression [212].

Recently, in a model of stress-induced aggression in Tph2^+/−^ mice, we have found alterations in Plp1 and myelin basic protein (Mbp) mRNA expression in the medial PFC [73]. In this study, stressed Tph2^+/−^ mice with increased aggressive behaviour revealed decreased expression of these genes [73]. In another model of stress (stress of “systemic inflammation” or “inflammatory stress”), in which elevated aggression in mice deficient for major brain gangliosides was observed, we also found decreased mRNA and protein expression of Plp1 in the medial prefrontal cortex of both male and female mice. These changes in myelin markers were associated with increased aggressive and dominant behaviour, although no alterations in *Plp1* expression were found in naïve non-stressed mutant mice [213].

Notably, there are recent data suggesting that myelination is dependent not only on oxidative stress but also, as mentioned above, on IR-mediated signalling. It was shown that oligodendrocytes in the mouse brain express both IR and insulin-like growth factor 1 receptor (IGF-1R) [214]. Although the functional significance of IRs in oligodendrocytes has not yet been studied, it was shown in the peripheral nervous system that the insulin resistance of Schwann cells (induced by Schwann cell-specific deletion of both IR and IGF-1R) leads to thinner myelin sheaths during development and adulthood [215]. The authors speculate that this effect stems from impairment of lipid metabolism via the phosphoinositide 3-kinase (PI3K)/protein kinase B (PKB)/mammalian target of rapamycin (mTOR) pathway [215]. Moreover, in an experiment with IR substrate-1 (IRS1) knockout mice, it was shown that overexpression of IGF-1 leads to increased mouse brain weight in IRS1 knockout and wild-type mice, although to a greater extent in wild type mice [216]. In a co-culture of glial cells and neurons, it was shown that overexpression of IGF-1 leads to an increase in Mbp and Plp1 content [216]. Together, these findings further suggest a crucial role for insulin signalling in the regulation of myelination and its potential role in the pathophysiology of MDD and associated aggression.

## 7. Challenges in the Management of Excessive Aggression 

Due to the fact that nowadays MDD is increasingly more frequently accompanied by pathological aggression, violence, self-harm, and suicide [4,16], this medical problem requires particular attention of physicians and researchers. To date, various classes of compounds are used in the pharmacotherapy of excessive aggression (Table 2). First of all, excessive aggression is usually not treated specifically but in conjunction with other psychiatric disorders such as MDD [217,218]. As has been discussed, the use of classic antidepressants is very common in patients suffering from excessive aggression associated with depressive symptoms. SSRIs are widely used clinically for the treatment of aggression, with fluoxetine probably one of the most used and studied drugs for this purpose [219,220,221]. Randomized clinical trials have shown that the non-selective beta-adrenoceptor and partial 5-HT1A receptor antagonists, propranolol and pindolol are effective for the management of aggression and agitation in patients with traumatic brain injury [222]. However, only large doses of these compounds were effective, with major side effects observed, such as bradycardia. Furthermore, the use of SSRIs in depressed patients can have an opposite effect on aggression, increasing these symptoms [14].

There are data showing a specific anti-aggressive effect of low doses of second-generation antipsychotic medications [243]. Risperidone, an atypical antipsychotic drug that blocks dopamine and 5-HT receptor systems, was shown to be effective for severe aggression in adolescents with disruptive behavioural disorders [244]. Most commonly, benzodiazepines (e.g., lorazepam) and antipsychotic medication are used to treat excessive aggression, either alone or in combination [245,246], but there is also evidence that in rare cases their administration may lead to increases in aggressive behaviour [247]. Furthermore, the use of benzodiazepines can aggravate the course of MDD. Hence, medications used in the treatment of excessive aggression often do not have sufficient therapeutic effect or specific effects on aggression and the currently available pharmacotherapy used in MDD patients (e.g., anticonvulsants) may have general sedative effects [248,249,250,251] and other side effects [252]. Drugs such as phenytoin [253] and valproate [254] were suggested for their effectiveness in the treatment of pathological aggression but may aggravate the symptoms of depression. The efficacy of antihypertensive drugs and psychostimulants was demonstrated in some cases of excessive aggression, but only marginal benefits were observed [255].

Based on the literature reviewed here, increased aggression is also associated with oxidative stress and neuroinflammation, which can be a potential target of pharmacotherapy for MDD patients suffering from uncontrollable aggressive behaviour. As such, the potential of antioxidant treatment in the management of aggression has been proposed in several studies [62,63,66,67,238]. Decreased levels of endogenous antioxidants, such as glutathione and superoxide dismutase, lead to an increase in oxidative stress, which in turn produces anxiogenic behaviour and aggression in mice [256]. Oxidative stress decreases expression of the MAOA gene, whose low activity has been implicated in violence and aggression [257]. The reactive oxygen species level was elevated in the brains of mice subjected to repeated forced swimming., but this increase was reversed using clomipramine, a tricyclic antidepressant [258]. Antioxidant and anti-inflammatory treatments are anticipated to be free from typical side effects of traditional anti-anxiety drugs and SSRIs [259]. Taking into account the above-reviewed data, it can be hypothesized that the use of compounds with anti-inflammatory and antioxidant properties may be a beneficial strategy for the depressive-like symptoms associated with excessive aggression and the accompanying molecular alterations.

## 8. New Strategies in Pharmacological Management of Pathological Aggression

As is indicated above, the recent literature reports the efficacy of various antioxidant and anti-inflammatory remedies in established rodent models of MDD and comorbid neuropsychiatric disorders with symptoms of anxiety, irritability and aggression [260] (Table 3). For example, ascorbic acid, beta carotene and vitamin E showed dose-dependent effects that significantly reduced the tail rattling, attacking and biting responses in an L-DOPA-induced aggression model [261]. In mice, treatment with lithium inhibited GSK-3 therefore has anti-inflammatory effects. This is associated with significantly reduced aggression, impulsivity and depression traits [262]. Moreover, the recent meta-analysis suggests the usefulness of antioxidant therapy, e.g., vitamin B and vitamin D in the management of depression, anxiety and accompanying symptoms [263,264,265]. It is suggested that these interventions should be considered as integral parts of MDD treatment, particularly in cases of its co-morbidity with substance use and alcohol dependence [169]. Another established therapy was proposed by clinical studies that revealed positive effects of the omega-3 fatty acids docosahexaenoic acid (DHA) and eicosapentaenoic acid (EPA), antioxidant and anti-inflammatory agents, in patients with depression and impulsivity [266,267]. Deprivation of dietary n-3 polyunsaturated fatty acid, a known antioxidant, has been found to increase both depression and aggressive behaviour in rats [268]. The observations with DHA and EPA were recently supported by combined clinical and pre-clinical study on adolescent depression.

An example of advantageous, well-tolerated, and risk-free antioxidant treatment of depressed patients and agitation is thiamine (vitamin B1) and its derivatives, whose administration was shown to exert beneficial effects on depressed patients [240,264,279]. Remarkably, chronic thiamine deficiency in a rat was found to induce muricide behaviour that was used to model aggression experimentally [280] and was supported by other studies [281]. By contrast, a treatment with thiamine compounds, such as thiamine, benfotiamine and dibenzoylthiamine, was shown to counteract stress-induced aggression and depressive-like manifestations [56,66,238,240,271].

The use of “insulin receptor sensitizers”, as it is mentioned above, appears to be an attractive solution, helping to reduce symptoms of depression [187]. Such effects were reported, e.g., for the rosiglitazone and pioglitazone, and other thiazolidinediones [282,283,284,285,286,287]. The latest translational studies have revealed possible mechanisms mediating these antidepressant-like effects, and demonstrated positive effects of anti-diabetic drugs on signs of depressive-like behaviour and pathological aggression [188,189,274,288,289,290]. 

Apart from the mentioned medical problems resulting from side effects of commonly used pharmacotherapy of agitated depression and aggression, the devastating economic situation in many societies can hamper the use of costly antidepressants and sedatives, particularly in countries with limited medical care. These factors necessitate the further development of inexpensive and effective alternatives to current therapies and prevention approaches [291,292]. Herbal medicine appears as a reasonable treatment of neuropsychiatric disorders which is more affordable and with fewer side effects than classic pharmaca.

Herbal medicine is known to normalize behavioural correlates of depressive-like state and stress-associated changes experimentally induced in small rodents. Herbal treatments with stress-reducing properties diminish inflammation and the production of free radicals, one of the mechanisms of distress [293,294] and aggression [295]. This was shown in mice receiving chlorogenic acid extract from *Prunus domestica* [296] or *Beta vulgaris* during restraint stress [297] and in rats treated with an extract from *Ulva sp.* [277]. However, with an increase in self-medication with herbal remedies, there is a need for better understanding of their mechanisms of action to prevent potential adverse effects and better control over a standardization of herbal compositions used for medicinal purposes [298]. 

A number of studies using standardized herbal cocktails reported the efficacy of chronic treatment with herbs that exerted antioxidant and anti-inflammatory effects [278,295]. For example, standardized herbal cocktail (SHC), an extract of clove, bell pepper, basil, pomegranate, nettle and other plants that was designed as an antioxidant treatment, was reported to reduce signs of increased depressive and aggressive behaviour in mice subjected to a model of ultrasound “emotional” stress, and in the paradigm of enhanced learning of adversities/PTSD [63]. This was accompanied by a normalization of brain oxidative markers and ameliorative effects of chronic administration of SHC on other stress-induced molecular read-outs [63]. Similarly, chronic administration of a standardized herbal composition containing seaweed, ginger, lemon, orange elderberries and other elements has normalized excessive aggression in BALB/c mice in the ultrasound stress model [62]. These effects were suggested to be due to the amelioration of hippocampal functions, such as malondialdehyde content, GSK3β, expression of pro-inflammatory cytokines Il-1β and Il-6, and the number of Ki67-positive cells, as well as the internalization of AMPA receptor subunits GluA1-A3 [62].

Finally, as medicinal herbs exert fewer side effects than conventional drugs and are affordable for low-income societies, the use of these kinds of remedies appears particularly beneficial for the improvement of mental health, including MDD and associated pathological aggression, under the conditions of the ongoing economic crisis. We suggest that standardized herbal compositions, vitamin B1 compounds and natural omega-3 consumption through the diet should be promoted. 

## 9. Conclusions

**Figure 1:**
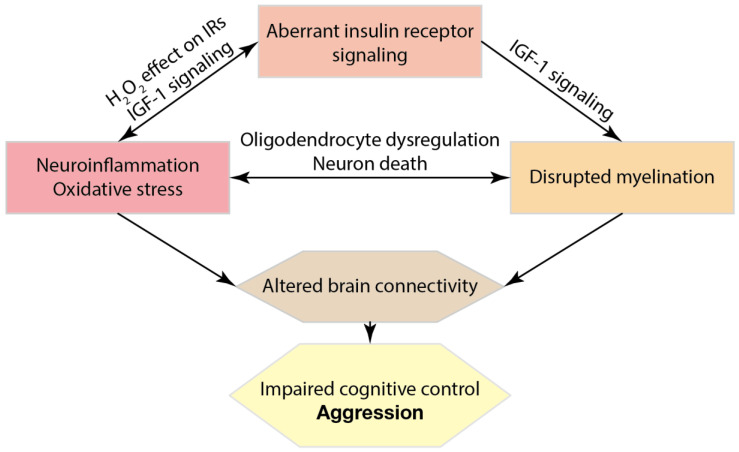
Pathological molecular pathways of neuroinflammation, myelination and insulin receptor signalling resulting in impaired cognitive control and excessive aggression in patients with depression. Major depression disorder and elevated stress response can be accompanied by neuroinflammation and oxidative stress, which affect brain insulin receptor (IR) signalling and myelination. The autophosphorylation of IR was shown to be highly sensitive to H_2_O_2_ signalling enhanced by oxidative stress [187]. It may alter IGF-1 signalling, where IGF-1 has neuroprotective and anti-inflammatory effects [299]. IGF-1 also promotes myelination via IRs on oligodendrocytes [214,216]. Impaired myelination might further trigger neuroinflammation as myelin debris, products of neuron elimination, and dysregulation of activity-dependent astrocytes are known to activate microglia and macrophages [300]. In turn, pro-inflammatory cytokines and dysregulation of glia by inflammation negatively affect myelination [301]. Aberrant signal propagation, axon degradation and neuronal death due to a lack of metabolic support from myelin sheaths can impair t brain connectivity, resulting in deficient cognitive control, e.g., disruption of cortical-subcortical connections, and thus contributing to excessive aggression.

During the last decade, the classic monoamine theory of depression and associated symptoms of MDD has been adjusted and extended considerably. An increasing body of evidence points to brain alterations occurring not only in classic neurotransmitters but also in neuronal connectivity that could be caused by disrupted myelination and triggered by an increase in oxidative stress and neuroinflammation. These alterations were observed both in clinical and animal studies of depressive disorder accompanied by pathological aggression. A recently hypothesized “triad” of inter-related molecular mechanisms of neuroinflammation, myelination and IR signalling might underlie the deficiency in brain connectivity contributing to the pathophysiology of MDD and impulsivity control in depressed patients (Figure 1). Consequently, this view suggests that compounds targeting oxidative stress, neuroinflammation and the activity of oligodendroglia may be considered as new approaches to offer more specific and effective treatment of the depressive symptoms associated with excessive aggression.

## Figures and Tables

**Table 2:** Pharmacotherapy of aggression associated with depressive syndrome. Effects of classical antidepressants and drugs with antidepressant-like effects are systematized by drug classes, abbreviations are: BDNF—brain-derived neurotrophic factor; GSK3-β—glycogen synthase kinase-3 beta; MAOI—monoamine oxidase inhibitor; NMDA—N-methyl-D-aspartate; SNRI—serotonin norepinephrine reuptake inhibitor; SSRI—selective serotonin reuptake inhibitor; TCA—tricyclic antidepressant.

Drug Class	Drug Examples	Strains	Core Targets	References
Benzodiazepine	Adinazolam Diazepam	Sprague-Dawley rats	Facilitating GABAergic transmission, decreasing neuronal excitability	[223,224]
TCA	Amitriptyline Desipramine Imipramine	Sprague-Dawley rats, Wistar rats, C57BL/6J mice	Blocking of serotonin transporter SERT and norepinephrine transporter NET, inhibition of sodium channels, reversed lipid peroxidation	[225,226,227,228]
SSRI	Citalopram Escitalopram Fluoxetine	Sprague-Dawley rats, Wistar rats	Inhibition of serotonin reuptake, increased norepinephrine transmission, upregulation of BDNF, anti-inflammatory effects	[229,230]
MAOI	Moclobemide	Sprague-Dawley rats	Inhibition of monoamine oxidase activity, deamination of serotonin and norepinephrine	[231]
NMDA antagonist	Ketamine MK-801	Wistar rats	Inhibition of ionotropic NMDA receptors, anti-inflammatory effects	[232,233,234]
SNRI	Duloxetine	Sprague-Dawley rats	Inhibition of serotonin and norepinephrine reuptake, antioxidant activity	[235]
Typical antipsychotic	Haloperidol	Wistar rats	Blocking of dopamine receptor type 2	[236,237]
Essential vitamins and their synthetic derivates	Thiamine Benfotiamine Dibenzoylthiamine Vitamin E	BALB/C, CD1, C57BL/6J mice	Antioxidant activity, increased neuroplasticity, overexpression of BDNF, anti-inflammatory effects, downregulation of GSK3-β, increased glutathione content	[56,62,66,238,239,240]
Insulin receptor sensitizers	Rosiglitazone Pioglitazone	BALB/C, CD1, C57BL/6J mice	Decreased neuronal damage, anti-inflammatory effects, increased mitochondrial biogenesis	[187,241,242]

**Table 3:** Preclinical studies addressing the use of new treatments of excessive aggression.

Treatment	Model	Effects on Aggression	Other Effects	References
Ascorbic acid Beta carotene Vitamin E N-acetyl cysteine	Isolation, or L-DOPA, male Swiss albino mice	↓	Increased levels of GSH, SOD, CAT in brain	[261]
Lithium chloride	Shock-induced, Sprague-Dawley rats; shock induced plus d-AMP or scopolamine, Walter Reed rats; isolation, AB mice; resident-intruder, TO mice	↓	—	[269]
	Shock-induced, CD-1, C57BL/6J and FVB/N mice	↓	Increased brain norepinephrine turnover	[270]
Thiamine Benfothiamine Dibenzoylthiamine	Ultrasound-induced, BALB/c mice	↓	Anxiolytic, anti-depressant, reduced inflammation and oxidative stress	[56,66,238,240,271]
Dicholine succinate	C57BL/6N mice; Western diet, C57BL/6 mice; chronic stress, CD-1 mice; defeat stress, C57BL/6J mice	↓ (pilot data)	Anxiolytic, anti-depressant, prevention of Tlr4 upregulation in brain	[188,272,273]
Rosiglitazone	Chronic social defeat, C57BL/6J mice	Not assessed	Anxiolytic, anti-depressant	[274]
Chlorogenic acid extract from *Prunus domestica* or *Beta vulgaris*	Swiss albino mice, alone or with restraint stress	Not assessed	Anxiolytic, anti-depressant, reduced ROS production by immune cells in vitro, increased GSH level and decreased MDA in brain tissue	[275,276]
*Ulva* sp. extract	Wistar rats	Not assessed	Anti-depressant	[277]
Extract of blackberry chamomile, garlic, cloves, and elderberry	Resiquimod- or LPS-induced inflammation, CD-1 mice	Not assessed	Anxiolytic, anti-depressant. Reduced expression of SAA2, ACE2, CXCL1, CXCL10, Il-1β, Il-6 in spleen and liver. Normalized counts of neutrophiles, monocytes, eosinophiles.	[278]
Standardized herbal cocktail (see [63] for composition)	Ultrasound stress, BALB/c and C57BL/6 mice	↓ (pilot data)	Anti-depressant. Decreased brain MDA and protein-carbonyl, decreased brain expression of IL-1β and IL-6	[63]
Standardized herbal cocktail (see [62] for composition)	Ultrasound stress, BALB/c mice	↓	Anti-depressant. Decreased brain expression of Il-1β, Il-6, TNF, GSK-3β. Increased expression of Ki67, decreased brain MDA	[62]

Abbreviations: GSH—glutathione, SOD—superoxide dismutase, CAT—catalase, Tlr4—tall-like receptor 4, ROS—reactive oxygen species, MDA—malondialdehyde. SAA-2—serum amyloid A, ACE-2—angiotensin-converting enzyme 2, CXCL1—chemokine ligand 1, CXCL10—C-X-C motif chemokine ligand 10, GSK-3β—glycogen synthase kinase 3 beta; ↓—a decrease.

## Data Availability

Not applicable.
